# Long-Term Oral Exposure to Chlorophenylacetonitrile Induces Gastrointestinal Morphological Alterations and Splenic Transcriptomic Reprogramming in Mice

**DOI:** 10.3390/toxics14080647

**Published:** 2026-07-23

**Authors:** Yayun Zhang, Fei Liu, Hao Zhou, Jingwen Chen, Lei Jiang, Changchun Yan, Xiaodong Li, Dingming Xue, Jiangfei Wang

**Affiliations:** 1 State Key Laboratory of Water Pollution Control and Green Resource Recycling, College of Environmental Science and Engineering, Tongji University, Shanghai 200092, China; 2 Shanghai Institute of Pollution Control and Ecological Security, Shanghai 200092, China; 3 Key Laboratory of Lake and Watershed Science for Water Security, Nanjing Institute of Geography and Limnology, Chinese Academy of Sciences, Nanjing 210008, China; 4 University of Chinese Academy of Sciences, Beijing 100049, China; 5 College of Nanjing, University of Chinese Academy of Sciences, Nanjing 211135, China; 6 Nanjing Institute of Environmental Sciences, Ministry of Ecology and Environment, No.8 Jiangwangmiao Street, Nanjing 210042, China; 7 Institute of Agricultural Facilities and Equipment, Jiangsu Academy of Agricultural Sciences, Jiangsu Engineering Technology Research Center of Biomass Composites and Addictive Manufacturing, Key Laboratory for Protected Agricultural Engineering in the Middle and Lower Reaches of Yangtze River, Ministry of Agriculture and Rural Affairs, Nanjing 210014, China; 8 Ecological and Environmental Monitoring Center of Zhejiang Province, Hangzhou 310012, China

**Keywords:** nitrogenous disinfection byproducts, 2-chlorophenylacetonitrile, gut–spleen axis, epithelial barrier failure, immunotoxicity, cell cycle arrest

## Abstract

Chlorophenylacetonitriles are known as one of the emerging nitrogenous disinfection byproducts (N-DBPs) in chlorinated drinking water due to their concerned cytotoxicity and genotoxicity compared to regulated carbonaceous DBPs. However, under low-dose exposure, the in vivo pathological consequences of chlorophenylacetonitriles remain largely unresolved. Here, C57BL/6J mice were exposed to 2-chlorophenylacetonitrile (2-CPAN) via drinking water (100 ug/L) for six months, and an integrated histopathological and genome-wide transcriptomic approach was employed to mechanistically characterize its multi-organ toxicological consequences. 2-CPAN ingestion significantly suppressed body weight (35.9 ± 2.0 g vs. 47.6 ± 11.6 g, *p* < 0.05) and induced severe gastroenteropathy—including gastric lamina propria inflammatory infiltration, intestinal villous blunting, crypt disorganization, transmural mononuclear infiltration, and abrogating epithelial barrier integrity. Intestinal barrier failure drove portal translocation of luminal PAMPs, potentially triggering splenic white pulp atrophy, red/white pulp boundary dissolution, and parenchymal changes consistent with fibrotic remodeling. Splenic RNA sequencing revealed a bipartite transcriptomic reprogramming: upregulated pathways were enriched in the ribosome, MAPK signaling, cytokine–cytokine receptor interaction, and chemokine signaling pathways. A proteotoxic stress module (*Hspa1a*, 9.4-fold; *Hspa1b*, 10.2-fold) and a chemokine effector hub (*Ccl2*, 3.43-fold; *Ccl5*, 2.9-fold; *Ccl19*, 2.58-fold; *Ccl21a*, 2.15-fold) were identified by the STRING network. Downregulated pathways converged on cell cycle suppression, with concurrent loss of *Ccne1*/*Ccne2* and *Cdc6* (G1/S block), *Ccnb1* and *Plk1* (G2/M arrest), and *Rrm2*/*Tars3*/*TrnM* (dNTP and aminoacyl-tRNA starvation), collectively forcing splenic lymphocytes into irreversible proliferative failure. These findings provide the first mechanistically resolved in vivo evidence that chronic 2-CPAN exposure drives a potential gut–spleen toxicological axis, underscoring the urgent need to incorporate organ endpoints by long-term exposure into N-DBP risk assessment.

## 1. Introduction

The provision of safe and clean drinking water is a cornerstone of global public health and modern civilization [1]. To mitigate the risk of waterborne infectious diseases, chemical disinfection processes such as chlorination, chloramination, and ozonation are universally employed [2]. Although these practices effectively eradicate pathogenic microorganisms, they inadvertently react with naturally occurring organic matter and bromide/iodide ions, generating a complex mixture of disinfection byproducts (DBPs) [3]. Over the past few decades, regulatory bodies worldwide have strictly enforced guidelines on conventional carbonaceous DBPs (C-DBPs), such as trihalomethanes and haloacetic acids, which has substantially altered drinking water treatment paradigms [4]. However, this regulatory shift and subsequent modifications in disinfectants have driven a critical transition toward the emergence of unregulated, nitrogenous DBPs (N-DBPs) [5]. In recent environmental and toxicological fields, N-DBPs have become an urgent scientific hotspot due to their widespread occurrence in finished water and their significantly higher mammalian cytotoxicity and genotoxicity compared to regulated C-DBPs [6].

Among these emerging N-DBPs, halogenated phenyl nitriles, specifically 2-chlorophenylacetonitrile (2-CPAN), have raised immense concern due to their stable chemical structure and robust persistence in water distribution systems [7]. Previous in vitro toxicological characterizations have consistently revealed that 2-CPAN possesses potentially unhealthy effects [8]. Despite these alarming cellular data, the in vivo pathological profiling of 2-CPAN remains profoundly understudied. The vast majority of existing literature is strictly limited to acute, high-dose threshold-driven toxicology, leaving a critical scientific blind spot regarding the physiological consequences of chronic, low-dose exposure through a relevant ingestion route [9]. Consequently, evaluating the long-term chronic hazard potential of 2-CPAN in vivo represents an indispensable gap that must be addressed for proper environmental risk characterization and population-level health assessments [10].

To unravel the systemic pathology of chronic 2-CPAN ingestion, we propose a mechanistic hypothesis centered on the “Gut-Spleen Axis” [11]. As the primary site for the luminal absorption of oral xenobiotics, the gastrointestinal (GI) tract is the first physiological line of defense and the most immediate target of waterborne chemical irritants [12]. Long-term exposure to reactive aromatic nitriles can disrupt the delicate mucosal epithelium, dismantle tight junction gatekeepers, and trigger local transmural inflammatory responses [13,14]. Critically, this sustained compromise of the GI physical barrier facilitates the systemic translocation of luminal chemical stressors, reactive metabolites, and gut-derived pathogen-associated molecular patterns (PAMPs) into the portal circulation [15]. The spleen, acting as the largest peripheral lymph organ and primary blood-filtering surveillance hub, is uniquely exposed to these blood-borne toxins [16]. Therefore, we hypothesize that chronic 2-CPAN exposure shatters distal splenic tissue homeostasis through a cascading gut–spleen narrative, bridging local enteropathy with global immunotoxicity [17].

To validate this multi-tiered pathological hypothesis, this study established a long-term (six-month) physiologically relevant oral exposure model in mice to mimic chronic environmental ingestion through contaminated water supplies. We deployed an integrative approach combining morphological and genome-wide transcriptomic analyses at the experimental endpoint. Specifically, we performed a thorough histopathological evaluation of the gastrointestinal tract (stomach and intestine) to quantify mucosal barrier compromise, alongside whole-transcriptome RNA sequencing (RNA-seq) of the spleen to map global immune reprogramming. By bridging observable tissue-level enteropathy with sophisticated splenic molecular networks, this work provides the first systematic in vivo evidence of chronic 2-CPAN toxicity, establishing a robust foundation for the toxicological risk assessment of emerging nitrogenous disinfection byproducts.

## 2. Materials and Methods

### 2.1. Chemicals and Reagents

2-Chlorophenylacetonitrile (2-CPAN, CAS No. 2856-63-5, purity ≥ 97%) was purchased from Sigma-Aldrich (St. Louis, MO, USA). Hematoxylin and eosin (H&E) staining kits were obtained from Solarbio Science and Technology Co., Ltd. (Beijing, China). All other chemical reagents used in this study were of analytical grade.

### 2.2. Animal Care Exposure Model

All animal experimental procedures were reviewed and approved by the Institutional Animal Care and Use Committee (IACUC) of Tongji University. Specific pathogen-free (SPF) male C57BL/6J mice (6–8 weeks old, weighing 20.5 ± 1.5 g) were obtained from Charles River Laboratories (Beijing, China). Animals were housed in standard polycarbonate cages under controlled environmental conditions (temperature: 22± 2 °C; humidity: 50 ± 10%; 12 h light/dark cycle) with ad libitum access to standard rodent chow.

Following a 1-week acclimation period, mice were randomly assigned to two experimental cohorts (n = 5): the control group and the 2-CPAN exposure group. The exposure group received 2-CPAN dissolved in their drinking water at a concentration of 100 ug/L continuously for six months (24 weeks) to simulate chronic environmental contamination [18]. The control group received similar drinking water. Fresh drinking water solutions were replenished every 3 days. Based on standard reference water consumption values for adult C57BL/6J mice (approximately 5 mL/day for a 25–30 g mouse) [19], the estimated daily intake of 2-CPAN was approximately 16.7 μg/kg body weight/day, which falls within an environmentally plausible range for chronic low-level exposure via contaminated drinking water. Individual body weights were monitored and recorded at regular weekly intervals throughout the 6-month exposure framework as an integrative indicator of systemic health and overall toxic burden.

### 2.3. Tissue Collection and Histopathological Examination

At the conclusion of the 6-month exposure period, all mice were fasted overnight and humanely sacrificed via beheading to ensure death. Tissues, including the stomach, small intestine, and spleen, were rapidly excised. For histopathological characterization, fragments of the stomach, intestine, and spleen were immediately fixed in 4% paraformaldehyde solution for 24–48 h, dehydrated through a graded series of ethanol, cleared in xylene, and embedded in paraffin blocks.

Paraffin-embedded tissues were sectioned at a thickness of 5 um using a rotary microtome. The sections were then mounted on glass slides and subjected to standard hematoxylin and eosin (H&E) staining according to established histological protocols [20]. Morphological changes, including gastric mucosal inflammation, intestinal villous height, crypt morphology, and splenic red/white pulp compartmentalization, were examined and captured under an advanced light microscope (Olympus BX53, Tokyo, Japan) equipped with a digital camera. Histopathological assessment was independently performed by two observers blinded to the experimental group allocation.

### 2.4. Total RNA Extraction and Quality Control

The remaining portion of the fresh splenic tissues was snap-frozen in liquid nitrogen and stored at −80 °C until downstream molecular processing. Total RNA was extracted from the frozen splenic parenchyma using RNAiso Plus (Takara Bio Inc., Shiga, Japan) according to the manufacturer’s instructions. The concentration and purity of the isolated RNA were determined using a NanoDrop 2000 spectrophotometer (Thermo Fisher Scientific, Wilmington, DE, USA). RNA structural integrity was strictly assessed using an Agilent 2100 Bioanalyzer (Agilent Technologies, Santa Clara, CA, USA). Only RNA samples with an RNA Integrity Number (RIN) ≥ 7.0 and an A260/A280 ratio between 1.8 and 2.0 were utilized for downstream RNA sequencing.

### 2.5. Genome-Wide RNA Sequencing (RNA-Seq) and Bioinformatics Analysis

Transcriptomic library construction and genome-wide sequencing were performed by Biotree Co., Ltd. (Shanghai, China). Briefly, poly(A) mRNA was purified from total RNA using oligo(dT) magnetic beads, fragmented into short pieces, and used as a template for first- and second-strand cDNA synthesis. The resulting double-stranded cDNA fragments underwent end-repair, A-tailing, and ligation with sequencing adapters. After size selection and PCR amplification, the qualified libraries were sequenced on an Illumina NovaSeq PE150 platform to generate 150 bp paired-end reads.

Raw data (raw reads) were filtered using Fastp (version 0.23.4) to remove low-quality reads, adapter sequences, and poly-N tags to obtain clean reads. The clean reads were then aligned to the mouse reference genome (GRCm39) using HISAT2 (version 2.2.1) [21]. Gene expression levels were quantified based on fragments per kilobase of transcript per million mapped fragments (FPKM) and clean read counts using FeatureCounts (version 2.0.6). Differentially expressed genes (DEGs) between the control and 2-CPAN-exposed spleens were identified using the DESeq2 R package [22]. Transcripts with an adjusted *p*-value (*P_adj_*) < 0.05 and an absolute log_2_ fold-change (|log2FC|) ≥ 1 were defined as significantly DEGs. KEGG pathway enrichment analysis of the DEGs was implemented using the ClusterProfiler R package [23], with a threshold of *P_adj_* < 0.05 designating statistical significance.

### 2.6. Protein–Protein Interaction (PPI) Network Topology

To analyze the functional interaction networks of the leading-edge differentially expressed transcripts, the Search Tool for the Retrieval of Interacting Genes/Proteins (STRING, version 12.0, string-db.org) database was queried [24]. A confidence score threshold of ≥0.400 was applied to construct the PPI topology. The network map was visualized and annotated for functional modules using Cytoscape software (version 3.10.1, cytoscape.org) to highlight key hub genes commanding biological cascades [25].

### 2.7. Statistical Analysis

All quantitative experimental data from weight monitoring and morphometric evaluation are expressed as the mean ± standard of the mean (SEM). Statistical comparisons between the vehicle control group and the chronic 2-CPAN-exposed group were conducted using the unpaired Student’s *t*-test via GraphPad Prism software (version 10.0, GraphPad Software, San Diego, CA, USA). A *p*-value < 0.05 was considered statistically significant.

## 3. Results

### 3.1. Experimental Setup and Rationale for the Chronic Oral Model

**Figure 1:**
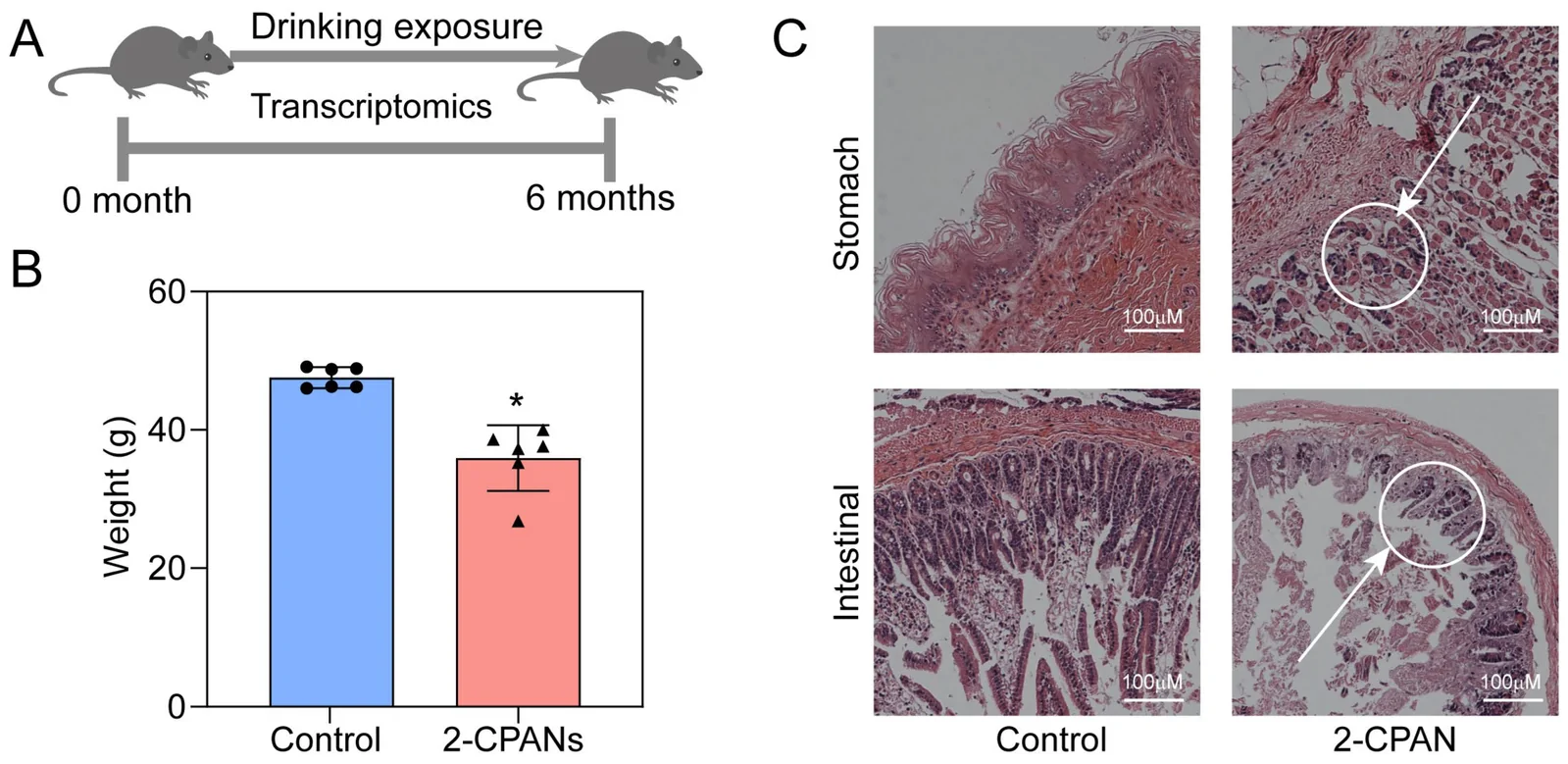
Global growth inhibition and multi-regional gastroenteropathy following long-term ingestion of 2-CPAN. (**A**) Experimental design for the evaluation of 2-CPAN toxicity via drinking water exposure in mice. (**B**) Statistical quantification of somatic growth and body weight at the experimental endpoint. (**C**) Histopathological characterization of the upper (stomach) and lower (intestinal) gastrointestinal tracts from the control and 2-CPAN exposure groups. White circles and arrows denote pathological foci, including glandular disruption and villous blunting, *: *p* < 0.05, ** *p* < 0.01.

We established a long-term oral exposure model in which mice received 2-CPAN dissolved in their drinking water over a defined period, with subsequent multi-organ histopathological and transcriptomic analyses performed at the experimental endpoint (Figure 1A). The drinking water exposure paradigm was selected because it closely approximates the scenario of chronic low-level environmental contamination through contaminated water supplies, which represents one of the most toxicologically relevant routes of human exposure to industrial nitrile compounds. Throughout the exposure period, all animals were maintained under standardized housing conditions with ad libitum access to food and medicated or vehicle-treated water, and body weight was monitored at regular intervals as a sensitive, non-invasive indicator of systemic health and overall toxic burden. At the conclusion of the exposure period, animals were sacrificed, and tissues, including the stomach, intestine, and spleen, were collected for downstream histopathological and molecular analyses.

This experimental design integrates morphological and transcriptomic endpoints to provide a comprehensive, multi-level characterization of 2-CPAN toxicity in vivo. By combining histopathological assessment of the gastrointestinal tract—the primary site of luminal xenobiotic absorption—with genome-wide splenic transcriptomics, the study was designed to capture both local and systemic consequences of chronic 2-CPAN ingestion and to bridge the gap between observable tissue pathology and underlying molecular mechanisms.

### 3.2. Suppression of Growth Kinetics and Terminal Body Weight

Body weight serves as one of the most reliable and integrative indicators of systemic toxicity in rodent models, reflecting the cumulative impact of a chemical insult on metabolic homeostasis, nutritional status, and overall organismal fitness. A comparison of terminal body weights between the two experimental groups revealed a marked and statistically significant reduction in the 2-CPAN-exposed cohort relative to vehicle-treated controls (Figure 1B). Control animals exhibited consistently high body weights with minimal inter-individual variation, as evidenced by the tight clustering of individual data points around the group mean, indicative of a healthy, physiologically stable population under normal conditions. In striking contrast, mice in the 2-CPAN-exposed group displayed substantially lower terminal body weights, with a mean value of approximately 35.9 ± 2.0 g compared with 47.6 ± 11.6 g in the control group (*p* < 0.05). The notable error bars observed in the 2-CPAN group suggest meaningful inter-individual variability in the growth suppression response, which may reflect differences in individual metabolic detoxification capacity, drinking water consumption rates, or intrinsic genetic susceptibility to 2-CPAN-mediated toxicity among the exposed animals.

#### 3.2.1. Inflammatory Infiltration and Glandular Disruption in Gastric Mucosa

To directly assess the histopathological consequences of chronic 2-CPAN ingestion on the primary site of xenobiotic absorption, hematoxylin and eosin (H&E) staining was performed on gastric tissue sections obtained from control and 2-CPAN-exposed animals (Figure 1C, upper panels). In vehicle-treated control mice, the gastric mucosa exhibited a well-preserved and orderly architecture across all histological layers. The surface epithelium was continuous and intact, comprising columnar mucous cells without evidence of erosion or desquamation. The glandular compartment was populated by regularly arranged gastric glands of uniform caliber, with distinct zonal organization into the isthmus, neck, and base regions. The lamina propria between glandular units contained sparse stromal cells and minimal immune infiltrate, consistent with the physiological baseline immune surveillance activity of the normal gastric mucosa. The muscularis mucosae and submucosal layers were intact and unremarkable.

In stark contrast, the gastric mucosa of 2-CPAN-exposed mice exhibited a constellation of histopathological abnormalities indicative of chemically induced chronic gastric injury. Most prominently, as highlighted by the circled regions and arrows in Figure 1C, dense aggregates of inflammatory cells were identified within the lamina propria and submucosal compartments, consistent with a robust innate immune response to sustained mucosal irritation. The glandular architecture was focally disrupted, with evidence of glandular dilation, irregular glandular contours, and, in some regions, loss of normal zonal organization, features collectively reminiscent of chronic active gastritis as defined by established histopathological criteria. The surface epithelium showed focal areas of thinning and nuclear irregularity, suggesting early epithelial injury and potential compromise of the mucus-secreting barrier function that normally protects the underlying tissue from luminal chemical insult. Taken together, these gastric histopathological findings are consistent with a chemically induced chronic mucosal inflammatory phenotype, most likely driven by direct irritant effects of luminal 2-CPAN or its reactive metabolites on the gastric epithelium, with secondary recruitment of innate immune effectors.

#### 3.2.2. Villous Atrophy and Epithelial Barrier Compromise in the Intestine

Histopathological examination of the small intestinal mucosa further demonstrated that chronic 2-CPAN exposure induces profound structural alterations at a second critical site of luminal xenobiotic absorption (Figure 1C, lower panels). In vehicle-treated control animals, the intestinal mucosa presented classic normal morphology: tall, finger-like villi of uniform height and width projected into the intestinal lumen, each covered by a continuous monolayer of columnar enterocytes with well-defined brush borders. Crypts of Lieberkühn were clearly defined structures of regular depth and consistent cellular composition, housing the progenitor cell populations responsible for continuous epithelial renewal. The lamina propria within the villi contained normal densities of stromal cells and immune cells, including resident macrophages and intraepithelial lymphocytes at physiological levels. The submucosal and muscular layers were intact, without edema or cellular infiltration.

Intestinal sections from 2-CPAN-exposed mice, however, revealed extensive and multifocal mucosal pathology. The most conspicuous finding was marked villous blunting, with qualitatively evident reduction in villous height relative to crypt depth—features indicative of compromised mucosal integrity and absorptive surface area—in the exposed group compared with controls. Individual villi appeared shortened, broadened, and in some areas fused, with irregular epithelial coverage and focal denudation of the villous surface. Crypt architecture was substantially disorganized, with evidence of crypt dilation, irregular crypt depth, and reduced mitotic activity, suggesting impairment of the proliferative compartment responsible for mucosal self-renewal. Critically, the lamina propria of exposed animals contained a markedly increased burden of inflammatory cells, including lymphocytes and mononuclear phagocytes, consistent with activated mucosal immunity in response to epithelial barrier compromise (Figure 1C, circled regions). The submucosal layer showed evidence of edema and focal mononuclear infiltration, indicative of a transmural extension of the inflammatory process beyond the epithelial compartment. These intestinal histopathological findings are consistent with a xenobiotic-induced enteropathy, sharing morphological features with previously described models of chemically induced villous atrophy and barrier dysfunction.

The convergence of inflammatory infiltration, villous atrophy, and crypt disorganization across both the stomach and intestine strongly indicates that 2-CPAN induces a sustained, multi-regional inflammatory response throughout the upper and lower gastrointestinal tract following chronic oral exposure.

#### 3.2.3. Splenic Follicular Atrophy and Parenchymal Fibrosis

**Figure 2:**
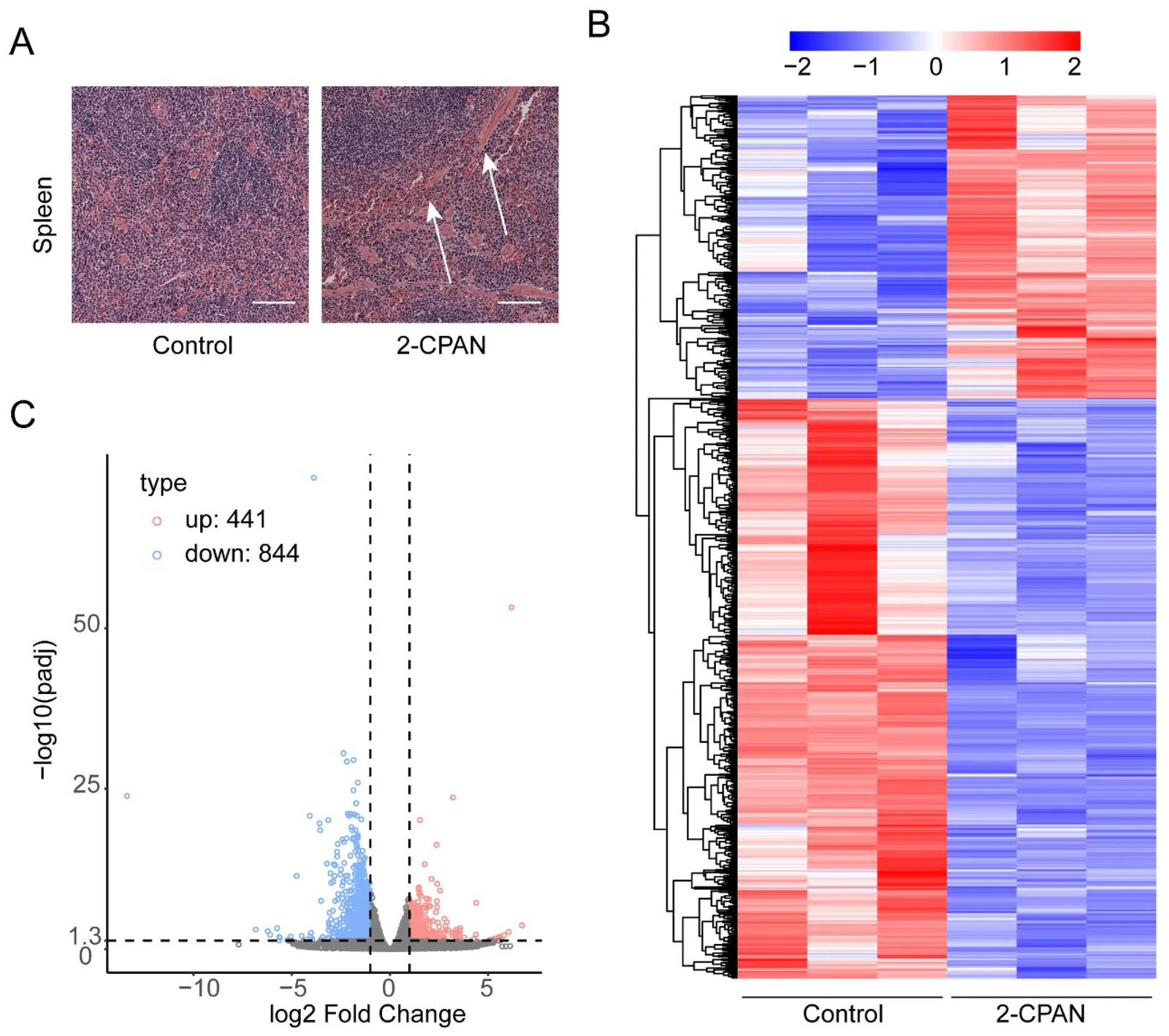
Histopathological pathology and macroscopic profiling of the splenic transcriptome under chronic 2-CPAN-induced stress. (**A**) Morphological characterization of the control and 2-CPAN-treated spleens, highlighting white pulp follicular atrophy and red/white pulp boundary dissipation. (**B**) Transcript expression profiles matrix visualized via heatmap, displaying within-group concordance and distinct inter-group expressional polarization. (**C**) Asymmetric distribution of up- and down-regulated DEGs represented by a volcano plot, indicating a predominant transcriptional suppression signature in the 2-CPAN group. Whight arrows: fibrous tissue deposition and hypocellular zones.

The spleen serves as the primary lymphoid organ responsible for systemic immune surveillance and the orchestration of adaptive immune responses. To determine whether chronic 2-CPAN ingestion perturbs splenic tissue homeostasis, histopathological examination of splenic sections was performed by hematoxylin and eosin (H&E) staining in control and 2-CPAN-exposed animals following six months of drinking water exposure (Figure 2A). In vehicle-treated control mice, splenic architecture was well-preserved, displaying distinct white pulp follicles with prominent germinal centers, clearly delineated marginal zones, and a richly cellular red pulp populated by erythrocytes and resident macrophages.

In contrast, splenic sections from 2-CPAN-exposed animals revealed profound and multifocal histopathological alterations. The white pulp follicular structures were notably atrophied, and their boundaries with the surrounding red pulp were indistinct. Large areas of parenchymal disruption were also observed, characterized by fibrous tissue deposition and hypocellular zones, as highlighted by the arrows in Figure 2A.

#### 3.2.4. Global Landscape and Reproducibility of the Splenic Transcriptome

To gain molecular insight into 2-CPAN-induced splenic pathology, RNA sequencing (RNA-seq) was performed on total RNA isolated from splenic tissue of control and 2-CPAN-exposed animals. Unsupervised hierarchical clustering of the resulting transcriptomic profiles, visualized as a heatmap of all differentially expressed genes (DEGs), revealed clear segregation of gene expression patterns between the two experimental groups (Figure 2B). The heatmap displayed a bipartite structure, with a cluster of genes showing markedly elevated expression (red) in 2-CPAN-exposed animals relative to controls and a corresponding cluster showing robust downregulation (blue) in the exposed group. Expression patterns within each group were concordant across biological replicates.

The transcriptomic perturbation induced by 2-CPAN spanned a broad range of gene ontology categories, including immune regulation, cellular stress responses, metabolic reprogramming, and structural remodeling. Volcano plot analysis, plotting log2 fold-change (log2FC) against the −log10-transformed adjusted *p*-value (−log10[*P*adj]), demonstrated an asymmetric distribution of DEGs, with the bulk of statistically significant genes displaced toward negative log2FC values (approximately −10 to −3), indicating a larger downregulated gene set relative to the upregulated cluster (Figure 2C, detailed information is shown in Table S1).

**Figure 3:**
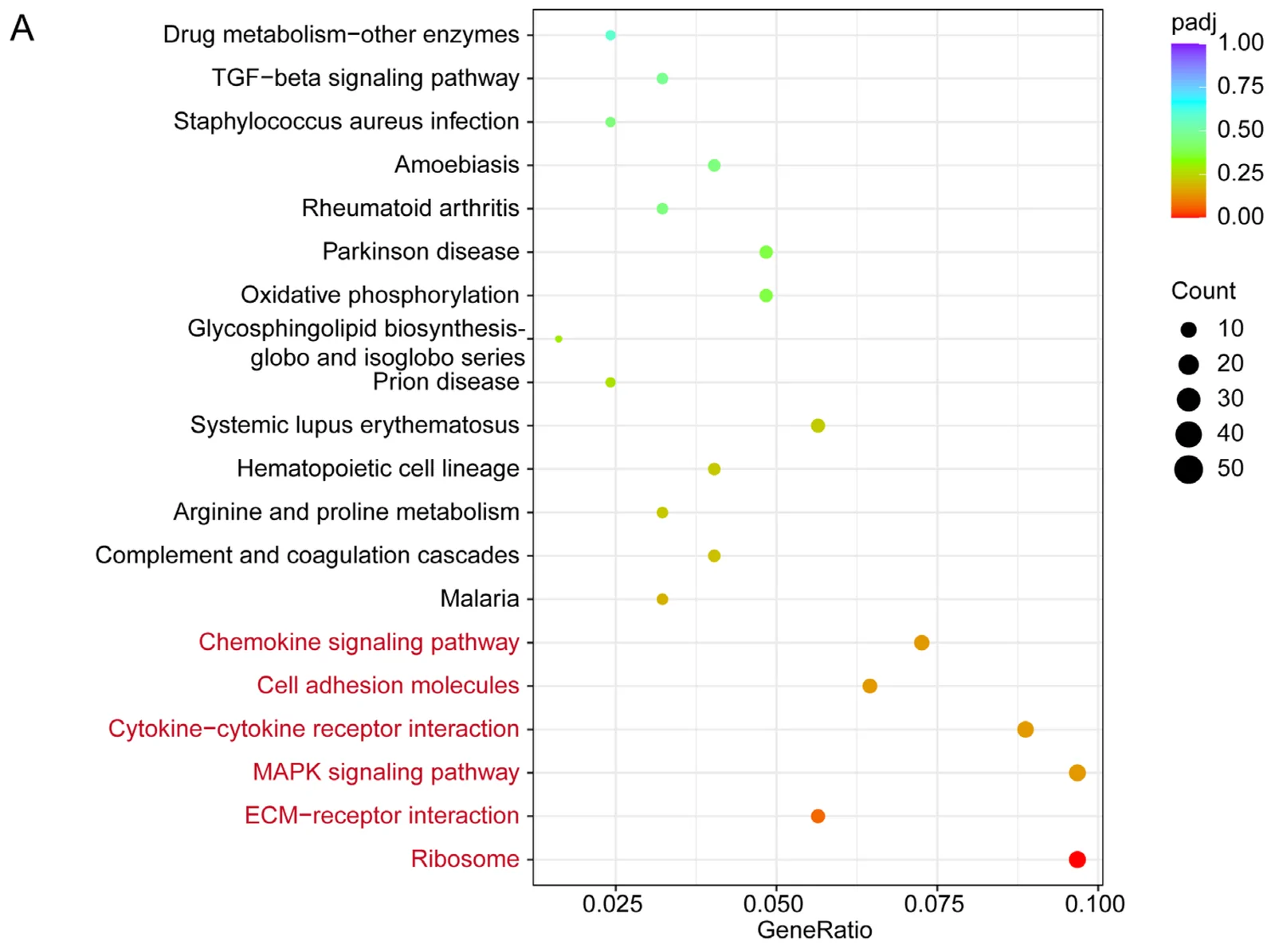

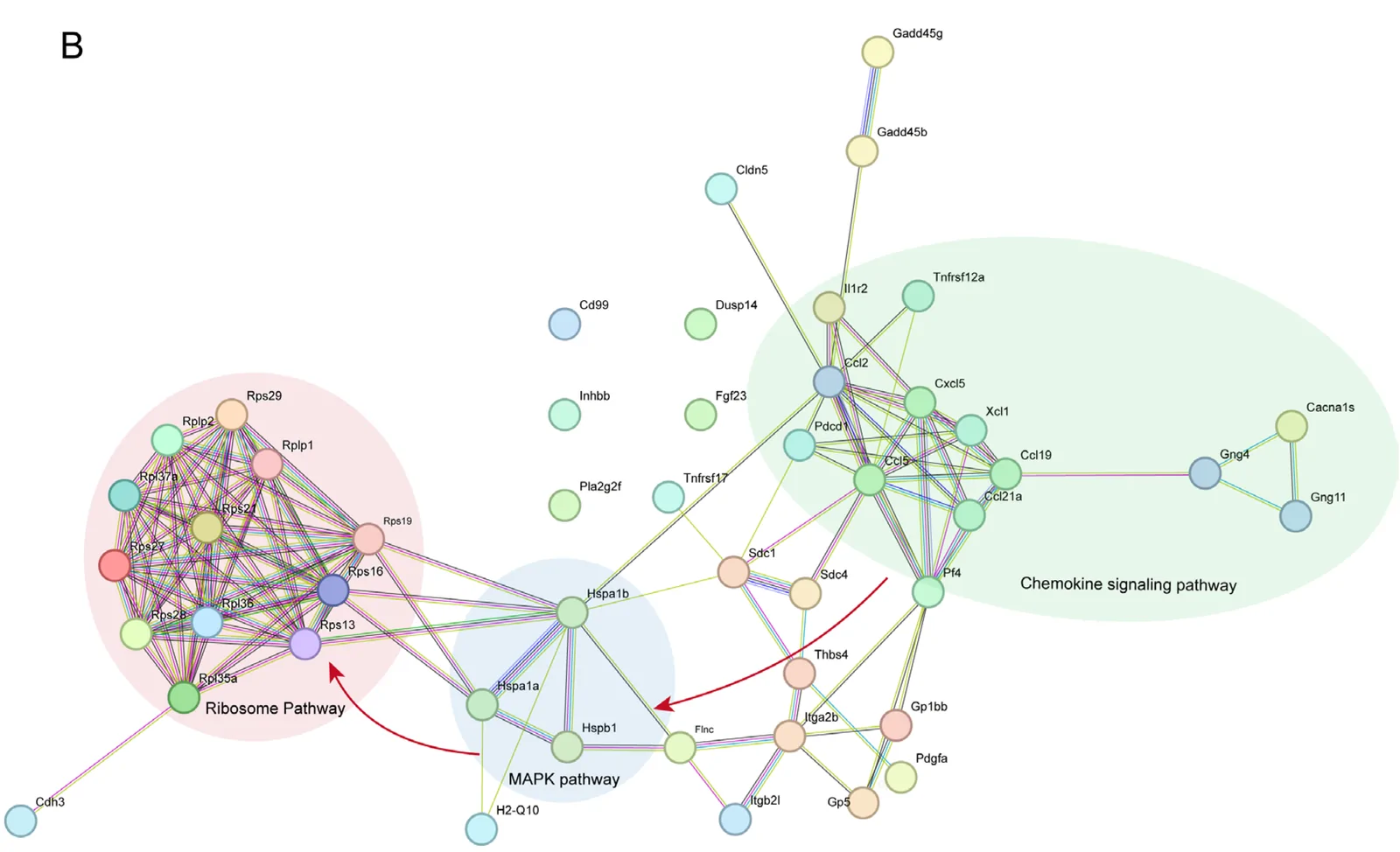
Coordinated upregulation of cellular stress, translational machinery, and inflammatory signaling networks post-2-CPAN exposure. (**A**) Enrichment dot plot visualizing the top-ranked KEGG biological pathways driving the splenic toxicological response, with *ribosome* and *MAPK signaling pathways* emerging as central pivots. padj refers to the *p* value adjusted for multiple testing using the Benjamini–Hochberg method (false discovery rate, FDR); a smaller padj indicates a more statistically significant pathway enrichment. (**B**) Topology of functionally clustered up-regulated DEGs. The network illustrates the spatial organization of ribosome biogenesis components, molecular chaperones (*Hspa1a/b*), and homeostatic chemokine coordinates (*Ccl2*, *Ccl5*, *Ccl19*, and *Ccl21a*), which structurally govern the splenic architectural remodeling and chronic localized inflammation.

To delineate the biological pathways preferentially activated in the spleen of 2-CPAN-exposed animals, KEGG pathway enrichment analysis was performed on the upregulated DEGs. The resulting enrichment dot plot (Figure 3A) visualizes the top enriched pathways ranked by FDR-adjusted *p*-value (dot color) and gene count (dot size). The identified pathways spanned immune activation, extracellular matrix remodeling, intracellular stress signaling, and ribosomal biogenesis. Among these, the ribosome pathway was the most statistically significant term, represented by the largest and most intensely colored dot, followed by ECM–receptor interaction, MAPK signaling pathway, cytokine–cytokine receptor interaction, cell adhesion molecules, and chemokine signaling pathway. An additional cohort of approximately twelve pathways, displayed as progressively smaller and lower-significance dots, was also detected.

#### 3.2.5. Transcriptional Activation of MAPK, Chemokine, and Ribosomal Stress Pathways

STRING database analysis was performed on the upregulated DEGs, and the resulting interaction network was rendered with functional module annotation (Figure 3B). The network revealed a structured topology comprising three principal interaction modules, connected by a small number of inter-module bridging nodes, along with several peripheral nodes reflecting less highly connected components of the response.

The first module, delineated by a dark red ellipse, constituted a densely interconnected ribosomal protein (RP) hub encompassing both small subunit (40S) and large subunit (60S) ribosomal protein-encoding genes, including *Rps19*, *Rps10*, *Rps13*, *Rps3*, *Rps32*, *Rps35*, *Rps29*, *Rpl37a*, *Rpl32*, *Rpl2*, and *Rpl39*, with virtually every node connected to every other by multiple edges reflecting co-expression, physical interaction, and database-curated co-pathway membership.

The second module, circumscribed by a dark blue ellipse, represented a proteostasis and heat shock response hub centered on *Hspa1a* and *Hspa1b*, together with *Hsb1* and *H2-Q10*. A directed edge connected *Rps13* (ribosomal module) to this HSP70 cluster.

The third module, enclosed within a dark green ellipse, comprised a chemokine and cytokine immune signaling hub organized around *Ccl5*, *Ccl2*, *Cxcl5*, and *Xcl1*, together with *Ccl10*, *Ccl21a*, *Il1r2*, and *Tnfsf12a*. Additional nodes within this module included *Gadd45a* and *Gadd45b* (connected by a high-confidence edge), *Ptk*, *Sod1*, *Thbs*, *Itga2b*, *Gp1bb*, *Pdgfa*, *Gnai4*, *Gnai1*, and *Casna1a*. A second directed edge connected *Fins* (fibronectin-related) to the ECM-associated gene cluster. Peripheral, less highly connected nodes included *Dusp14*, *Inhbb*, *Fg23*, *Pla2g7*, *Tnfrsf17*, *Cd69*, and *Cd9*.

**Figure 4:**
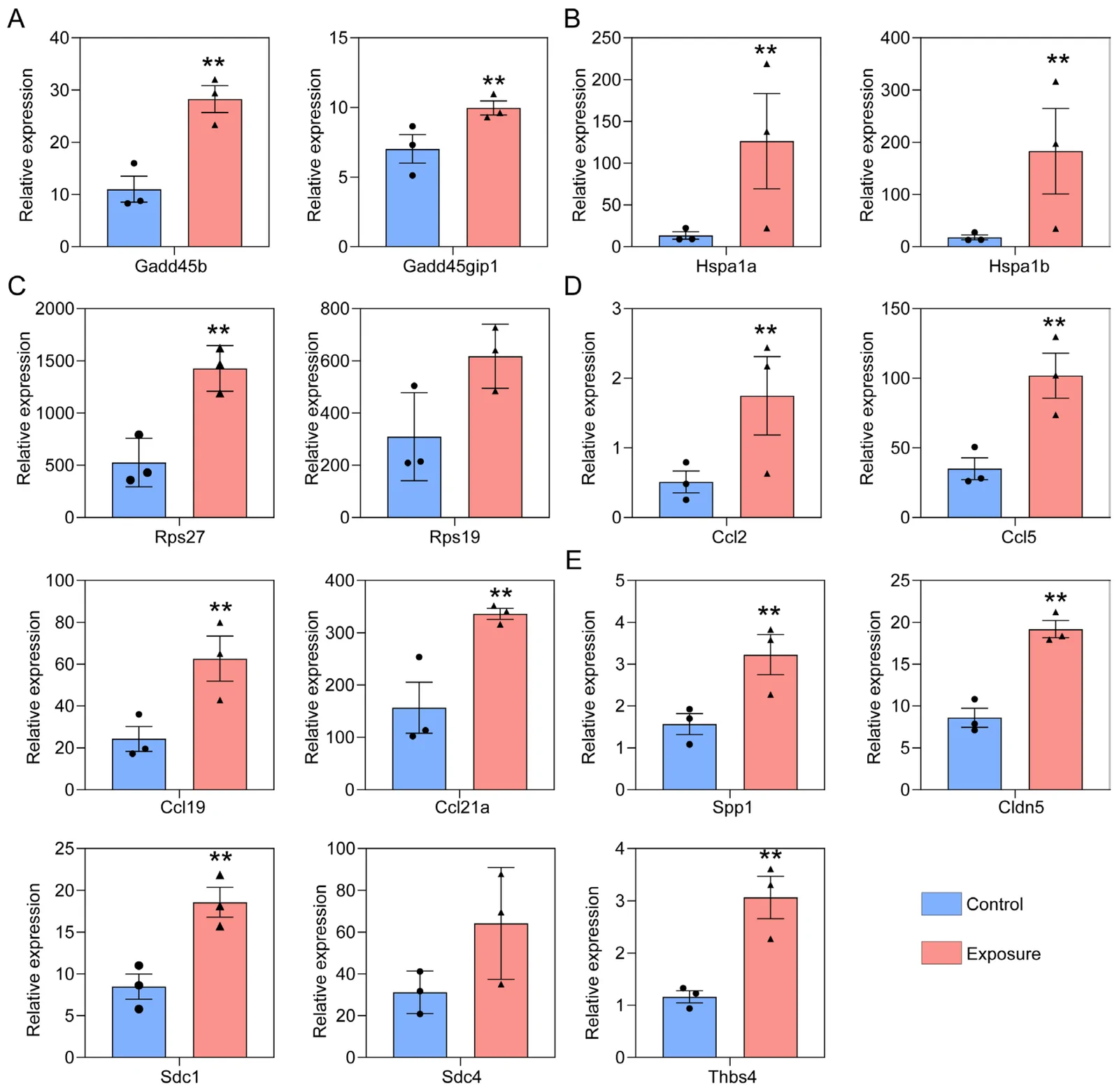
Normalized RNA-seq expression levels of representative upregulated differentially expressed genes. (**A**) DNA damage/stress-responsive genes (*Gadd45b*, *Gadd45gip1*); (**B**) heat shock proteins (*Hspa1a*, *Hspa1b*); (**C**) ribosomal protein genes (*Rps27*, *Rps19*); (**D**) chemokine signaling genes (*Ccl2*, *Ccl5*, *Ccl19*, *Ccl21a*); (**E**) extracellular matrix and cell adhesion-related genes (*Spp1*, *Cldn5*, *Sdc1*, *Sdc4*, *Thbs4*). Blue bars represent the control group; red bars represent the 2-CPAN-exposed group. Data are presented as mean ± SEM (n = 3 per group), *: *p* < 0.05, ** *p* < 0.01.

To further examine the expression of leading-edge genes identified by KEGG and STRING analyses, normalized RNA-seq expression levels of representative genes from each functional module were extracted and compared between groups (Figure 4). Within the MAPK signaling cascade, *Gadd45b* was upregulated approximately 2.6-fold, while its downstream interactor *Gadd45gip1* exhibited a consistent upward trend (Figure 4A). The molecular chaperones *Hspa1a* and *Hspa1b* were upregulated 9.4-fold and 10.2-fold, respectively (Figure 4B). Within the ribosome pathway, *Rps27* increased from 526.04 ± 115.30 (control) to 1426.70 ± 223.10 (exposed), and *Rps19* expression approximately doubled (Figure 4C). Among chemokine signaling genes, *Ccl2* and *Ccl5* were upregulated 3.43-fold and 2.9-fold, respectively, while *Ccl19* and *Ccl21a* were upregulated 2.58-fold and 2.15-fold, respectively (Figure 4D). Among ECM–receptor and cell adhesion molecule genes, *Cldn5* increased 2.24-fold, the proteoglycans *Sdc1* and *Sdc4* increased 2.19-fold and 2.06-fold, and the matrix remodeling genes *Spp1* and *Thbs4* increased 2.4-fold and 2.6-fold, respectively (Figure 4E).

#### 3.2.6. Deficits in DNA Replication and Insurmountable Cell Cycle Arrest

**Figure 5:**
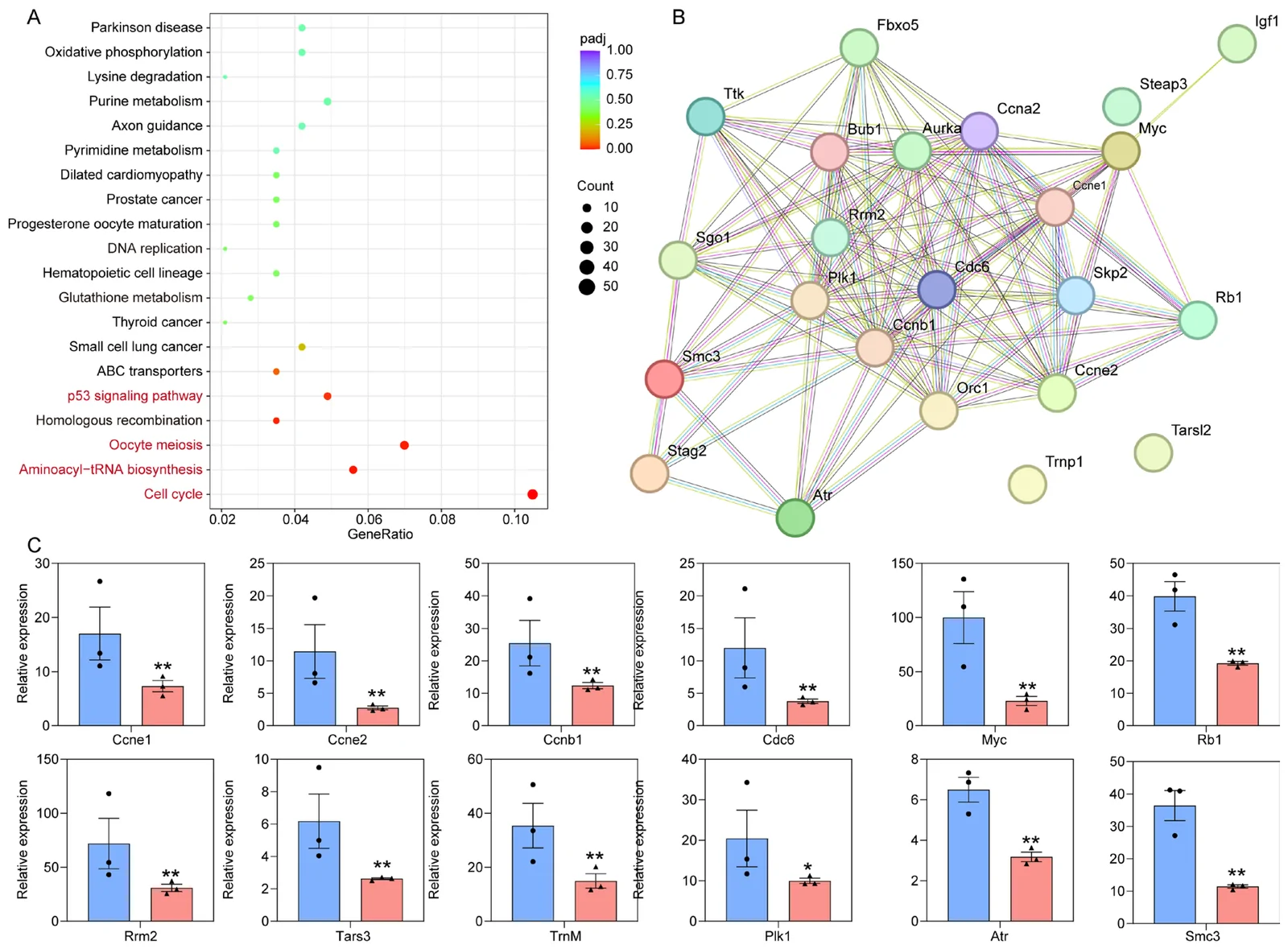
Mass suppression of cell cycle engines, DNA replication machinery, and aminoacyl-tRNA biosynthesis post-2-CPAN exposure. (**A**) Enrichment dot plot visualizing the top-ranked down-regulated biological cascades, with the cell cycle and aminoacyl-*tRNA biosynthesis* standing as the most heavily suppressed functional entities. (**B**) Disruption of the central replication-mitosis network topologically visualized by a PPI map, indicating a generalized transcriptomic shutdown of proliferation programs. (**C**) Grouped quantitative comparison of representative down-regulated genes, cross-validating the irreversible proliferation block and translational starvation within the splenic parenchyma, *: *p* < 0.05, ** *p* < 0.01. circles means the control group, triangles means the exposure group.

For functional consequences of spleens, the down-regulated transcriptomic profiles were comprehensively interrogated (Figure 5). Global KEGG pathway enrichment revealed that the most potently suppressed biological entities were predominantly localized to replication, translation, and cell division networks, commanded by the cell cycle (the most significantly enriched entity with GeneRatio > 0.10), aminoacyl-tRNA biosynthesis, Oocyte meiosis, and the p53 signaling pathway (Figure 5A). To further dissect the topological hierarchy of these alterations, a protein–protein interaction (PPI) network was constructed (Figure 5B). The network mapping revealed an intensely interwoven cluster of hub nodes, wherein critical cell cycle drivers, checkpoint kinases, and DNA licensing factors (*Ccnb1*, *Plk1*, *Ccne1*, *Cdc6*, and *Rrm2*) stood as the central regulatory core potentially contributing to the toxicological phenotype.

To cross-validate this profound transcriptional shutdown, targeted quantitative validation was conducted on the core components identified in the PPI and KEGG networks (Figure 5C). In perfect alignment with the global suppression of cell proliferation, the initiation and progression of the cell cycle were suggestive of multiple critical checkpoints. Specifically, *Ccne1* and *Ccne2*, the essential rate-limiting cyclins driving the G1-to-S phase transition, were markedly suppressed, alongside a drastic reduction in *Cdc6*, a cornerstone element for pre-replication complex assembly and DNA licensing (Figure 5C). The downregulation of these upstream licensing components was synchronized with a prominent crash in *Myc* transcription (collapsing from a baseline of ~100 to less than 25) and the stable suppression of *Rb1* (Figure 5C), suggesting a complete deactivation of the mitogenic proliferative program.

## 4. Discussion

### 4.1. Central Pathological Framework: The Gut–Spleen Axis

This study provides the first systematic in vivo characterization of the chronic oral toxicity of 2-chlorophenylacetonitrile (2-CPAN), a persistent aromatic nitrogenous disinfection byproduct (N-DBP) detected in finished drinking water [26]. Our multi-tiered evidence validates a cascading “gut–spleen toxicological axis” as the central pathological framework. Chronic 2-CPAN ingestion disrupts the gastrointestinal mucosal barrier, enabling the systemic translocation of luminal stressors and pathogen-associated molecular patterns (PAMPs), which subsequently drive distal splenic immune dysregulation and lymphocyte proliferative failure. Notably, these systemic outcomes are inaccessible to traditional in vitro toxicological characterizations, underscoring the necessity of in vivo long-term bioassays [27].

### 4.2. Gastrointestinal Cytotoxicity and Barrier Dysfunction

The significant body weight suppression in exposed animals is multifactorially driven by reduced nutrient absorption secondary to intestinal villous atrophy, catabolic cytokine signaling, and potential xenobiotic-induced anorexia. Histopathologically, the gastric mucosa exhibited dense inflammatory infiltration and epithelial thinning indicative of chronic gastritis. The intestinal injuries were more severe, featuring marked villous blunting and crypt disorganization, which are hallmark phenotypes of xenobiotic-induced enteropathy where progenitor self-renewal fails to compensate for accelerated epithelial loss [28]. These regional lesions are plausibly driven by the direct cytotoxicity of 2-CPAN or its reactive electrophilic metabolites generated via cytochrome P450-mediated nitrile bioactivation [29]. Mechanistically, we hypothesize that the observed villous atrophy and crypt disorganization may compromise tight junction integrity and increase paracellular permeability, potentially transforming the gut into a ‘leaky barrier’ that permits the systemic escape of endotoxins; this mechanism warrants direct validation in future studies [30].

### 4.3. Splenic Inflammation and Architectural Dissolution

As the primary vascular filter for blood-borne antigens, the spleen is uniquely vulnerable to gut-derived translocated signals, which may explain the observed splenic pathology following an orally administered compound without direct organ contact, although direct evidence of PAMP translocation was not obtained in this study [31]. The observed follicular atrophy and parenchymal fibrosis are consistent with chronically sustained inflammatory stimulation converging on the splenic microenvironment, likely driven by TGF-β-mediated stromal fibroblast activation [32].

At the transcriptomic level, this inflammatory storm is sustained by the coordinated hyperactivation of the MAPK and chemokine signaling pathways. The upregulation of *Gadd45b* links 2-CPAN-induced genotoxic stress to the inflammatory program via p38 MAPK [33]. Concurrently, elevated *Ccl2* and *Ccl5* recruit a multi-lineage inflammatory infiltrate. Most critically, the aberrant overexpression of homeostatic chemokines *Ccl19* and *Ccl21a* dismantles the CCR7-mediated spatial segregation of T and B lymphocyte zones, directly accounting for the histological dissolution of the red/white pulp demarcation [34].

### 4.4. Proteostatic Stress and Transcriptional Signatures of Proliferative Impairment

The transcriptomic profile reveals a pronounced proteotoxic stress signature, marked by upregulation of chaperones *Hspa1a*/*Hspa1b* (HSP70) alongside ribosomal protein genes (*Rps27*, *Rps19*), consistent with a cellular stress response to electrophilic metabolite burden, though this bulk-tissue signature may also partly reflect a shift in splenic cellular composition rather than uniform upregulation within cells [35]. This signature co-occurred with transcriptional changes suggestive of cell cycle arrest and substrate limitation in splenic lymphocytes.

Downregulation of *Ccne1*/*Ccne2*, *Cdc6*, and *Rb1* is consistent with a G1-phase blockade signature, while reduced *Ccnb1* and *Plk1* suggest an additional G2/M checkpoint signature [36]. Concurrent downregulation of *Rrm2* (rate-limiting for dNTP synthesis) and *Tars3* points to potential nucleotide and amino acid precursor limitation, which may contribute to impaired lymphocyte renewal under continued exposure [37].

*Ccnb1*, a key M-phase regulator, was significantly downregulated—a pattern commonly associated with G2/M arrest at the transcriptional level—alongside reduced *Plk1* and the DNA damage sensor *Atr*, suggestive of transcriptional changes linked to mitotic dysregulation. Reduced expression of the cohesin *Smc3* further suggests possible impairment of chromosomal structural integrity, though direct cytogenetic validation was not performed.

This cell cycle signature co-occurred with evidence suggestive of translational and metabolic limitation: sustained *Rrm2* downregulation may restrict dNTP availability for DNA synthesis and repair, while reduced *Tars3* and *TrnM* (tRNA-Met) suggest possible compromise of translational elongation and initiation capacity. Collectively, these transcriptomic findings provide converging molecular evidence consistent with a model in which chronic 2-CPAN exposure may drive splenic lymphocytes toward cell cycle arrest, impaired replication, and reduced translational output—a profile consistent with the white pulp atrophy observed histologically. Direct functional validation (e.g., flow cytometry, proliferation/apoptosis assays, phosphoprotein analysis) was not performed and is warranted in future work.

### 4.5. Limitations and Environmental Risk Implications

Several limitations warrant acknowledgment. This study employed a single 2-CPAN concentration; future dose–response studies will help establish NOAEL/LOAEL thresholds across environmentally relevant ranges. Histopathological evaluation, though performed in a blinded manner, was qualitative, and the proposed gut–spleen mechanistic link was supported by convergent histopathological and transcriptomic evidence rather than direct functional assays; incorporating single-cell sequencing and protein-level validation would further extend these mechanistic insights. Finally, while the sample size (n = 5 for histopathology/body weight; n = 3 for RNA-seq) is consistent with common practice in exploratory toxicological studies, larger cohorts would further strengthen statistical power in future work.

## 5. Conclusions

In summary, this study provides the first systematic in vivo evidence characterizing the chronic health risks of the emerging nitrogenous disinfection byproduct 2-chlorophenylacetonitrile (2-CPAN) under a physiologically relevant, long-term drinking water paradigm. Our multi-tiered morphological and transcriptomic findings validate a cascading “gut-spleen toxicological axis,” wherein chronic 2-CPAN ingestion is localized in gastrointestinal mucosal and vascular barriers that may facilitate translocation of chemotoxic stressors into distal circulatory surveillance hubs. Within the spleen, this unresolved irritation triggers intense MAPK inflammation and compensatory ribosomal proteostasis stress but ultimately causes irreversible replication failure, substrate starvation, and severe cell cycle arrest in lymphocytes. These insights advance our mechanistic understanding of DBP-mediated systemic immunotoxicity and underline the urgent necessity of incorporating chronic oral multi-organ evaluations into environmental safety assessments and drinking water quality guidelines.

## Data Availability

The original data presented in this study can be found in the NCBI public repository (accession number: PRJNA1495908). Further inquiries can be directed to the corresponding author(s).

## Supplementary Materials

The following supporting information can be downloaded at https://www.mdpi.com/article/10.3390/toxics14080647/s1; Table S1: gene expressions of spleens in mice.
